# Repeated partial splenic artery embolization for hypersplenism improves platelet count

**DOI:** 10.1515/med-2022-0479

**Published:** 2022-04-25

**Authors:** Youwen Tan, Jiamin Wang, Li Sun, Yun Ye

**Affiliations:** Department of Hepatology, The Third Hospital of Zhenjiang Affiliated Jiangsu University, Zhenjiang 212003, Jiangsu Province, China

**Keywords:** partial splenic embolization, platelet count, side effect, splenic abscess, liver function

## Abstract

Splenic embolization is a minimally invasive alternative to splenectomy for the treatment of hypersplenism. This was a retrospective study of 101 patients with hypersplenism caused by cirrhosis who were treated with splenic embolization and for whom 6 months of follow-up data were available. Of these patients, 65 underwent partial splenic artery embolization (PSE), including 23 who underwent repeated PSE (RPSE). The incidence of abdominal pain was significantly higher in the PSE group than in the total splenic artery embolization (TSE) group (*P* < 0.001), and its duration was also longer in the PSE group (*P* = 0.003). Biochemical markers of liver function were compared before and after the operation; aminotransferase indices decreased (alanine aminotransferase, aspartate aminotransferase, and alkaline phosphatase), total bilirubin increased slightly, and albumin and prealbumin decreased after the operation (all *P* < 0.001). Platelet (PLT) counts began to increase at 1 week postoperatively, peaked at 1 month postoperatively, and then decreased gradually. There was no significant intergroup (PSE and TSE) difference at any time point (1 day, 1 week, 1 month, and 6 months postoperatively, *P* > 0.05). There was a significant intergroup (PSE and RPSE) difference in the mean postoperative change in PLT count (*P* = 0.45). Splenic embolization can improve the inflammatory indicators of liver function. Performing PSE twice or more improves the PLT counts.

## 1 Introduction

Hypersplenism is one of the most serious complications of liver cirrhosis [1]. Splenic embolization is a minimally invasive procedure that provides an alternative to splenectomy for the treatment of hypersplenism [2]. Splenic artery embolization has fewer complications and is controllable, and the immune system is not significantly damaged because of sufficient retention of the splenic tissue. It has been widely used because of its minimal invasiveness [3]. Either total splenic artery embolization (TSE) or partial splenic artery embolization (PSE) may be performed [4,5]. The influence of the degree of splenic embolism on PSE efficacy remains controversial. The reduction in platelet (PLT) and white blood cell (WBC) counts caused by hypersplenism can be significantly improved when the proportion of splenic embolization is >50%, and its curative effect is further improved as it increases [6]. However, some scholars believe that there is no significant difference in the therapeutic effect when the proportion of splenic embolism is 50, 70, or 80% [7]. If PSE is performed and is not initially effective, the procedure may be repeated twice or more. Numerous studies to date have examined the benefit of splenic artery embolization prior to splenectomy [3,8,9,10,11]. However, there is insufficient research on the treatment of hypersplenism with repeated PSE (RPSE) for improving the hematology, liver function indices, and safety of hypersplenism.

In this study, we attempt to fill this gap in knowledge by observing outcomes and side effects over a prolonged period in patients with hypersplenism treated with splenic embolization.

## 2 Method

### 2.1 Ethics statement

This study was approved by the Medical Ethics Committee of The Third Hospital of Zhenjiang Affiliated Jiangsu University, and written informed consent was obtained from each patient prior to operation. This study was conducted in accordance with the Declaration of Helsinki.

### 2.2 Patients and parameters

**Figure 1:**
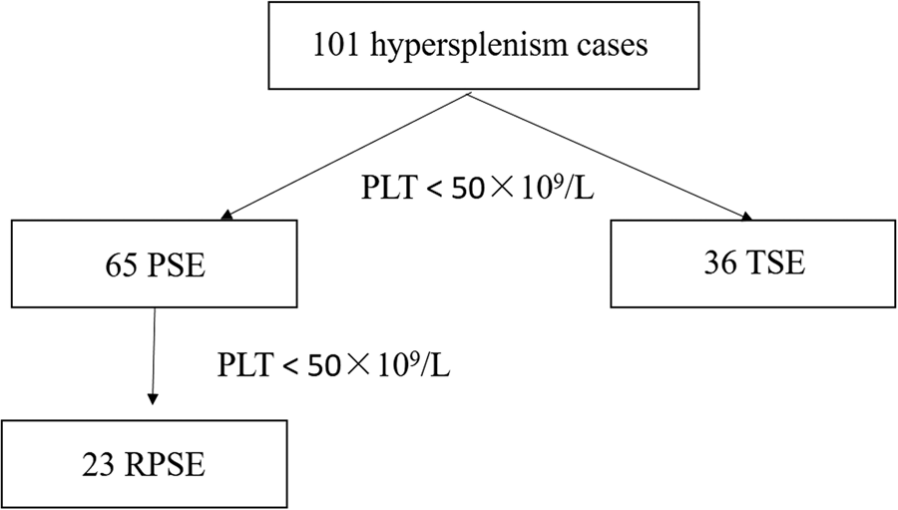
A flowchart of the study.

This study included 101 patients with hypersplenism caused by cirrhosis in our hospital center who were treated with splenic embolization between January 2011 and April 2021; all had a complete follow-up data for 6 months. Sixty-five patients underwent PSE, including 23 who underwent it twice or more PSE (RPSE); the other 36 underwent TSE (Figure 1). Biochemical markers of liver function (total bilirubin [TBIL], alanine aminotransferase [ALT], aspartate aminotransferase [AST], albumin [ALB], anti-lymphocyte globulin [ALG], prealbumin [PreALB], alkaline phosphatase [ALP], and gamma-glutamyl transpeptidase [GGT]) were evaluated preoperatively and 1 month postoperatively. Hematological indices (red blood cell [RBC] count, hemoglobin [Hb], mean corpuscular volume [MCV], WBC count, neutrophil count [NC], and PLT count) were evaluated preoperatively and at 1 day, 1 week, 1 month, and 6 months postoperatively. The incidence and duration of side effects (fever and abdominal pain) and the incidence of severe complications (splenic abscess and deep venous thrombosis) were also monitored. None of the patients were vaccinated against pneumococcal sepsis.

### 2.3 PSE method

**Figure 2:**
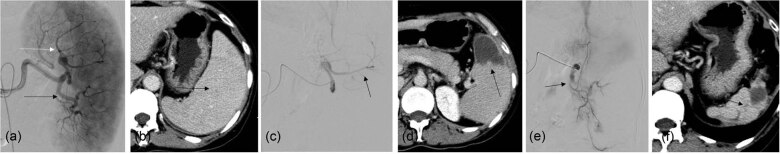
A 65-year-old woman with hepatitis B cirrhosis and hypersplenism underwent twice PSE. (a) Digital arteriography showed superior splenic artery branch (arrow) and inferior pole splenic artery branch (hollow arrow). (b) Splenomegaly (arrow). (c) First embolization of superior splenic artery (arrow). (d) One month after the first PSE, low-density infarct area was found in spleen (arrow). (e) The second PSE embolized the inferior splenic artery (arrow). (f) One year later, the spleen shrank (arrow).

PSE was carried out under strict aseptic conditions. After percutaneous puncture of the femoral artery, a 5 F catheter sheath and angiographic catheters were inserted. With the aid of a guide wire, the angiographic catheters were introduced into the abdominal trunk and splenic artery trunk to stain the splenic artery branches and splenic parenchyma, permitting determination of their size. Next, embolic material was introduced into the splenic artery via superselective catheterization. To avoid the dorsal pancreatic artery and short gastric artery, the material was slowly injected into the distal part of the main trunk or into the branches, following the low-pressure flow-control protocol. The embolic material consisted of gelatin sponge particles (1–2 mm) or polyvinyl alcohol particles (200–700 μm), which were mixed with gentamicin (16 mg), dexamethasone (8 mg), and a small amount of contrast agent (Figure 2).

The inclusion criteria were as follows: (1) clinical diagnosis of hypersplenism secondary to liver cirrhosis; (2) receiving PSE treatment; and (3) PLT count <50 × 10^9^/L. The exclusion criteria were as follows: (1) extrahepatic blood flow; (2) portal vein thrombosis (PVT); (3) combined systemic diseases such as primary immune thrombocytopenia; (4) end-stage liver disease; and (5) liver transplant recipients. The judgment of PSE or TSE was based on the operator’s surgery of catheter injection of embolic agent into the branch or total trunk of the splenic artery. RPSE was performed at a PLT count <50 × 10^9^/L after 1 month.

### 2.4 Statistical methods

All data were analyzed using SPSS 22 software (SPSS Inc., Chicago, IL, USA). Count data were recorded as numbers and percentages, and measurement data were recorded as means ± standard deviations. The independent sample *t* test and chi-square test were used to compare clinical data between the PSE and TSE groups. The paired sample *t* test was used to compare liver function before and after the operation. Single-factor repeated measures analysis of variance (ANOVA) with pairwise comparisons was used to compare hematological indices before and after the operation. Two-factor repeated measures ANOVA with pairwise comparisons was used to compare preoperative and postoperative hematological indices between the PSE and TSE groups as well as patients who underwent PSE once and those who underwent PSE twice or more.

## 3 Results

### 3.1 General clinical data

We examined 101 cases of liver cirrhosis complicated by hypersplenism. The cause was hepatitis B virus infection in 68 cases, hepatitis C virus infection in 25 cases, and schistosomiasis of the liver in 8 cases. The average patient age was 54.70 ± 8.47 years. There were 49 men and 52 women; 65 patients underwent PSE, and 36 underwent TSE. Postoperatively, 65 patients developed a fever (mean duration 4.25 ± 6.57 days), and 79 developed abdominal pain (mean duration 5.91 ± 6.68 days). There were 2 cases of splenic abscesses and 14 cases of deep vein thrombosis. The general clinical data of the PSE, RPSE, and TSE groups are shown in Table 1. The incidence of abdominal pain was significantly higher in the PSE group than in the TSE group (*P* < 0.001), and its duration was also longer in the PSE group (*P* = 0.003).

**Table 1:** Clinical features of PSE and TSE

Variables	Data			Vable	*P*
	PSE (*n* = 42)	RPSE (*n* = 23)	TSE (*n* = 36)		
Age	54.75 ± 8.46	54.21 ± 8.32	54.61 ± 8.59	0.183	0.855
Sex					
Men	16 (40%)	10 (43.5%)	23 (63.9%)	5.466	0.065
Women	26 (60%)	13 (56.5%)	13 (36.1%)		
Fever					
No	10 (23.8%)	9 (39.1%)	17 (47.2%)	4.79	0.091
Yes	32 (76.2%)	14 (60.9%)	19 (52.8%)		
Fever last time	4.78 ± 7.34	4.468 ± 7.11	3.18 ± 4.61	1.418	0.159
Abdominal pain					
No	4 (9.8%)	3 (13%)	15 (41.7%)	12.771	**0.002**
Yes	37 (90.2%)	20 (87%)	21 (58.3%)		
Abdominal pain time	7.25 ± 6.54	7.55 ± 6.76	4.0 ± 5.79	5.654	**0.004**
Splenic abscess					
No	41 (97.6%)	22 (95.7%)	36 (100%)	1.426	0.49
Yes	1 (2.4%)	1 (4.3%)	0 (0%)		

Bold values: *P* < 0.05.

### 3.2 Liver function

Biochemical markers of liver function were compared before and after the operation. As shown in Table 2, there were significant differences in the levels of most markers (TBIL, ALT, AST, ALB, ALG, PreALB, and ALP), except GGT. Among them, aminotransferase indices decreased (ALT, AST, and ALP), TBIL increased slightly, and ALB and PreALB decreased after the operation.

**Table 2:** Comparison of liver function before and after splenic embolization

	Pro-SE	Post-SE	*t*	*P*
TBIL	23.48 ± 11.72	29.97 ± 10.71	5.729	**<0.0001**
ALT	40.96 ± 29.83	25.31 ± 11.89	5.958	**<0.0001**
AST	45.34 ± 27.50	34.46 ± 13.82	4.07	**<0.0001**
ALB	37.97 ± 5.95	34.76 ± 4.61	8.714	**<0.0001**
ALG	31.54 ± 7.76	30.26 ± 7.63	3.593	**0.001**
PreALB	136.01 ± 64.42	88.96 ± 35.45	9.617	**<0.0001**
ALP	115.86 ± 77.73	107.38 ± 57.09	2.374	**0.02**
GGT	65.22 ± 67.51	65.93 ± 75.16	0.163	0.871

SE: splenic embolization. Bold values: *P* < 0.05.

### 3.3 Hematological indices (preoperative vs postoperative)

Hematological indices were measured at five different time points before and after the operation, as shown in Table 3. There were significant differences in each indices before and after the operation. RBC counts and Hb levels were lower at 1 day and 1 week postoperatively. WBC counts and NC increased rapidly from 1 day to 1 week postoperatively but had gradually decreased by 1 month postoperatively and continued to do so by 6 months. PLT counts began to rise 1 week after the operation, peaked 1 month after the operation, and then decreased gradually.

**Table 3:** Comparison of blood cell index before and after splenic embolization

	Pro-SE	Post-1D	Post-1W	Post-1M	Post-6M	*F*	*P*
RBC	4.11 ± 0.69	3.91 ± 0.58*	3.81 ± 0.66*	4.16 ± 1.84	4.17 ± 0.86	10.642	**<0.001**
HB	120.88 ± 2.59	117.67 ± 2.08	114.34 ± 2.37*	119.3 ± 3.42	123.87 ± 3.53	9.197	**<0.001**
MCV	88.49 ± 7.28	85.13 ± 7.38*	87.77 ± 7.21	87.70 ± 7.24	89.56 ± 8.24*	8.84	**<0.001**
WBC	3.03 ± 1.13	7.28 ± 3.38*	7.08 ± 6.34*	4.16 ± 1.84*	3.64 ± 1.75*	41.29	**<0.001**
NC	2.89 ± 1.39	5.79 ± 2.85*	5.47 ± 4.37*	3.37 ± 1.58*	2.37 ± 1.64*	36.44	**<0.001**
PLT	36.31 ± 17.17	52.86 ± 28.05*	83.46 ± 61.27*	73.17 ± 30.12*	66.53 ± 25.25*	35.01	**<0.001**
MPV	16.60 ± 1.69	11.92 ± 1.74*	11.39 ± 1.25*	10.77 ± 1.30*	11.25 ± 1.45*	5.987	**0.003**
HMT	0.043 ± 0.023	0.057 ± 0.022*	0.094 ± 0.063*	0.087 ± 0.035*	0.082 ± 0.042*	12.63	**<0.001**
LPR	39.78 ± 9.13	43.12 ± 10.92	36.76 ± 10.14	35.62 ± 7.97	35.54 ± 6.53*	4.084	**0.028**

*Compared with before treatment, *P* < 0.05.

SE: splenic embolization; RBC: red blood cell count; HB: hemoglobin; MCV: mean corpuscular volume; WBC: white blood cell count; NC: neutrophil count; PLT: platelet count; MPV: mean platelet volume; HMT: hematocrit; LPR: large platelet ratio. Bold values: *P* < 0.05.

### 3.4 Hematological indices (TSE vs PSE)

**Figure 3:**
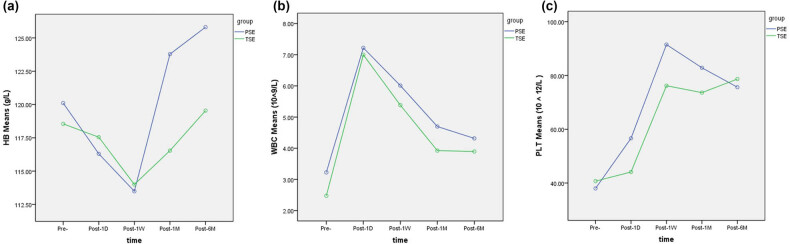
Changes of blood cells before and after PSE and TSE. (a) Changes of HB before and after PSE and TSE. (b) Changes of WBC counts before and after PSE and TSE. (c) Changes of PLT counts before and after PSE and TSE. HB: hemoglobin; MCV: mean corpuscular volume; MPV: mean platelet volume; HMT: hematocrit; LPR: large platelet ratio.

Hematological indices in TSE and PSE groups were measured at five time points before and after the operation, as shown in Table 4. It can be seen that there were significant differences in each indices before and after both PSE and TSE. There was no significant difference between the two groups at any time point. PLT decreased by 1 month somewhat less in the TSE group at 6 months postoperatively (Figure 3); however, there was no statistically significant difference between the groups.

**Table 4:** Comparison of hematological index between before and after splenic embolization

	Pro-SE		Post-1D		Post-1W		Post-1M		Post-6M		Group		Time	
	PSE	TSE	PSE	TSE	PSE	TSE	PSE	TSE	PSE	TSE	*F*	*P*	*F*	*P*
HB	120.43 ± 20.86	116.1 ± 34.54	117.45 ± 18.39	117.95 ± 19.91	114.28 ± 19.64	116.95 ± 19.57	124.47 ± 20.52	124.38 ± 20.17	130.18 ± 23.91	118.00 ± 31.14	0.241	0.627	3.927	**0.01**
WBC	3.19 ± 1.21	2.46 ± 1.54	7.58 ± 3.16	6.44 ± 3.23	5.89 ± 2.38	5.32 ± 2.09	4.65 ± 1.61	3.94 ± 1.72	4.30 ± 1.77	3.21 ± 1.59	3.396	0.074	65.796	**<0.001**
NC	2.39 ± 1.32	1.56 ± 0.72	5.93 ± 2.86	4.98 ± 2.88	4.02 ± 2.21	3.85 ± 1.79	3.10 ± 1.51	2.42 ± 1.26	3.25 ± 1.78	2.11 ± 1.42	2.472	0.125	48.336	**<0.001**
PLT	43.62 ± 26.15	38.76 ± 12.81	60.67 ± 42.15	41.64 ± 12.77	107.25 ± 76.92	87.11 ± 72.81	79.57 ± 50.96	81.56 ± 52.15	72.89 ± 31.65	76.75 ± 38.03	1.541	0.226	35.357	**<0.001**

Bold values: *P* < 0.05.

### 3.5 Hematological indices (single PSE vs RPSEs)

A total of 23 patients received PSE a second time (Figure 2). As shown in Table 5, there were significant differences in Hb and PLT before and after each PSE operation. As shown in Table 6, there was a significant difference between the first and second PSE operations with regard to the mean postoperative change in PLT.

**Table 5:** Comparison of hematological index between PSE and RPSE

	Pro-SE		Post-6M		Group		Time	
	PSE	RPSE	PSE	RPSE	*F*	*P*	*F*	*P*
HB	104.14 ± 23.47	115.34 ± 28.86	117.35 ± 27.86	118.45 ± 33.25	12.32	**<0.001**	13.236	**<0.001**
WBC	3.05 ± 1.53	3.26 ± 1.79	3.27 ± 1.75	3.36 ± 1.67	0.473	0.563	2.364	0.647
NC	2.14 ± 1.55	2.18 ± 2.01	2.35 ± 2.53	2.36 ± 1.75	0.113	0.887	1.532	0.886
PLT	46.65 ± 22.75	51.74 ± 25.76	48.25 ± 26.32	59.53 ± 38.03	6.375	**0.002**	5.754	**0.045**

SE: splenic embolization. Bold values: *P* < 0.05.

**Table 6:** Comparison of hematological index between first and second mean difference splenic embolization

	Mean difference		*t*	*P*
	PSE	RPSE		
HB	11.64 ± 5.23	4.13 ± 6.58	1.365	0.156
WBC	0.26 ± 0.21	0.21 ± 0.24	0.743	0.641
NC	0.18 ± 0.32	0.24 ± 0.12	1.041	0.113
PLT	3.64 ± 2.15	7.76 ± 2.76	3.554	**0.005**

Bold values: *P* < 0.05.

## 4 Discussion

In 50–64% of cases, portal hypertension caused by liver cirrhosis leads to splenic hyperemia, fibrosis, and hypersplenism. According to the literature [12], severe hypersplenism (with PLT counts less than 75 × 10^9^/L and WBC counts less than 2 × 10^9^/L) poses an increased risk of esophageal variceal bleeding and death. In addition, it has been reported that PLT counts less than 5 × 10^9^/L can increase the incidence of internal bleeding in autoimmune diseases [13]. Patients with abnormal hematological indices have a significantly increased 5-year incidence of decompensation of liver function and consequent mortality [14]. However, not all cases of hypersplenism require treatment. Nevertheless, correction of hypersplenism can increase blood cell counts, correct coagulation function, and provide an opportunity for traumatic treatment [15].

Splenectomy is a routine treatment for liver cirrhosis and hypersplenism. The main complications of splenectomy are postoperative infection and PVT. A prospective study found that the incidence of PVT after splenectomy was about 12.3% [16,17]. Owing to its serious and complex complications and the importance of the spleen for immune function, splenectomy is now discouraged if less invasive alternatives are viable.

Some researchers believe that PSE can improve liver function [18,19,20,21]. In a study of 101 patients conducted by Ishikawa [18], 53 patients simultaneously underwent transarterial chemoembolization (TACE) and PSE, while 48 patients underwent TACE alone. In the former, the Child–Pugh liver function deteriorated briefly after the operation and improved after 2 weeks. Lee et al. [20] reported that, at 2 months after PSE, the liver function of three of four patients improved from grade B to A. The improvement of liver function by PSE is mainly based on the reduction of portal vein pressure and the slowing down of liver fibrosis. This phenomenon was observed in our study. The important parametersof liver function (TBIL, ALT, AST, ALB, ALG, PreALB, and ALP), except for GGT. Our study also found that transaminase levels (ALT, AST, ALP) can be improved, a finding that is consistent with those of previous studies [20,21]. TBIL levels significantly improved after the operation. The transient rise in TBIL may be due to RBC destruction by PSE. The main manifestation is an increase in the indirect bilirubin level [22]. However, indicators reflecting the important synthesis and reserve functions of the liver did not improve. This may be because, although splenic embolism improves the inflammatory indicators of liver function, splenic embolism as an emergency factor is not beneficial to the original liver cirrhosis. This conclusion is not consistent with those of other studies [23].

Several studies of patients with hypersplenism due to hepatic cirrhosis have confirmed that PSE can relieve decreased PLT and WBC counts in the peripheral blood vessels and achieve good medium- and long-term outcomes [21,24,25,26]. Zaitoun et al. [27] showed that patients who underwent splenectomy for cirrhosis of the liver and preoperative PSE had less intraoperative blood loss and did not require surgical transfusion. We can also see from the rapid decline in RBC count and Hb level 1 day after the operation that PSE can temporarily cause RBC destruction. Another clinical follow-up study found that at 6 months after splenectomy, most patients showed varying degrees of increases in ALB levels, cholinesterase levels, total cholesterol levels, and liver volume [23]. In patients with liver cirrhosis and liver cancer who have hypersplenism, performing splenectomy at the same time as liver tumor resection can improve the restoration of liver function, and relieving hypersplenism can also improve liver function in patients with hepatitis C [28,29]. Studies have shown that after PSE, the blood stolen from the celiac trunk by the spleen decreases, the blood flow of the hepatic artery and superior mesenteric artery increases, and the oxygen supply and nutrition of the liver cells are improved [30].

In this study, we monitored changes in hematological indices at five time points before and after the operation. RBC counts and Hb levels decreased for a short time and gradually returned to the preoperative level by 1 month after the operation. In patients who received PSE, Hb levels did not significantly change from preoperative levels; these results are consistent with the literature [31]. WBC and NC increased rapidly after surgery and then decreased gradually, although they were still higher than preoperative levels at 1 month and 6 months. PLT is the main indices used to determine the efficacy of PSE. PLT counts increased rapidly and reached the peak at 1 week after surgery, gradually decreased over the next few weeks and months, and were still higher than the preoperative levels after 6 months.

PSE and TSE have also been compared in a previous study (27 patients underwent TSE and 34 patients underwent PSE). During the follow-up period, ranging from 6 months to 4 years after the operation, the former had higher WBC and PLT counts, a shorter hospitalization stay, and fewer complications. This study concluded that TSE was the better choice [32]. In our study, no significant difference was found in the increase in blood cell counts. Splenic artery embolization often features complications such as post-embolization syndrome, splenic abscess, splenic rupture, and gastrointestinal bleeding [33,34] as reported in our and many other studies [35,36,37,38,39]. Post-embolization syndrome, the most common minor complication, is usually well tolerated and controlled by conservative treatment [39,40]. The postoperative abdominal pain in patients with PSE is more obvious than that in patients with TSE. We believe that this phenomenon is because TSE is not a complete splenic infarction. TSE can be improved by the collateral arteries, which connect the left gastric artery or gastroepiploic artery to the splenic portal artery and provide a small amount of blood to the spleen, so as to avoid complete splenic infarction. PSE, especially splenic artery embolization in the lower pole, can lead to complete splenic infarction in the lower pole. Therefore, the postoperative abdominal pain was more obvious. In contrast, major complications such as splenic abscess have shown a close relationship between the incidence of complications and the rate of splenic infarction, and higher incidence of complications occurs in patients with splenic embolism >50% [19,34,41,42,43]. In our study, there were two cases of splenic abscess in the PSE group. For TSE, these did not occur, although the intergroup difference was not significant. The occurrence of splenic abscesses is also related to complete splenic infarction in the lower pole.

N’Kontchou et al. stated that as one-time embolization of an enlarged spleen may increase the incidence of complications and a splenic embolism can lead to a rapid decrease in the numbers of blood cells, such as PLTs, due to the active regeneration of the spleen, it may be necessary to conduct more embolizations [6]. In our study, 23 patients were treated with a secondary splenic embolization after the first splenic embolization failed to produce an ideal increase in blood cell count. The second embolization was generally able to further increase the number of blood cells, especially PLTs.

The limitations of this study are as follows: First, it was a retrospective study with a small sample size. Second, the larger efficacy of PSE is related to embolization volume, which is difficult to accurately measure, as well as the operator technique, which was not fully considered.

In conclusion, splenic embolization can partially improve the liver function indices. PSE and TSE demonstrate similar improvements in hematological indices, but the PLT-oriented indices soon decline after surgery, and the second PSE is conducive to improve the PLT count.
